# Legal assessment of ingrown horns and other horn-related anomalies in cattle and sheep

**DOI:** 10.1017/awf.2024.5

**Published:** 2024-01-29

**Authors:** Johanna Andersson, Johan Beck-Friis, Sirkku Sarenbo

**Affiliations:** Faculty of Health and Life Sciences, Department of Biology and Environmental Science, Linnaeus University, Kalmar, Sweden

**Keywords:** animal welfare, cattle, horn overgrowth, ingrown horns, sheep, suffering

## Abstract

Cattle and sheep horns have the potential to grow in such a way that the horn bends toward the animal’s head and, if left untreated, may penetrate the skin, causing pressure, pain, and suffering. According to the Swedish Animal Welfare Act, animals must be looked after in a way that prevents ingrown horns; otherwise, the person responsible for the animal may be prosecuted. Here, we present a review of 32 legal cases that occurred in Sweden between 2008 and 2022 for which the charge involved horn-related anomalies in cattle or sheep. The aim being to investigate the nature of these horn-related anomalies and the circumstances under which they occur. Of the legal cases, 53% were discovered during official animal welfare control on farms and 44% at an abattoir during pre-slaughter inspection. These include extreme injuries, e.g. both horns penetrating the periosteum into the skull bone, or a horn penetrating into the eye or oral cavity. The reasons offered by the accused for failing to detect animals with horn-related anomalies included that the animal appeared normal, that it was long-haired, shy, or hard to reach, or that the horns had not undergone gradual growth but had accidentally or suddenly penetrated the skin. Overall, 81% of the cases led to convictions; however, none of these resulted in imprisonment. Reasons for acquittals included insufficient crime description or evidence as to how the horn-related anomaly occurred or of the animal being exposed to suffering. A number of recommendations are provided that could help limit the occurrence of ingrown horns.

## Introduction

In domesticated, horned ruminants, the size and shape of horns are highly variable (e.g. Knierim *et al.* 2015) and, in sheep, even the number of horns varies among breeds (Anjola & McEwan 2018). In horned animals, horn size is an important determinant of social status (Nowak *et al.* 2008), and horns may also play a role in thermoregulation (Dwyer 2008; Knierim *et al.* 2015) as well as protection against predators (Aldersey *et al.* 2020). Cattle and sheep do not shed their horns in the same way as deer and elk, and their horns are very strong and resistant to extreme loading impacts, comparable in many ways to bulletproof glass (Tombolato *et al.* 2010). In cattle and sheep, depending on breed, both sexes can be horned or polled, and in certain sheep breeds, only the males are horned (Dwyer & Goddard 2022). In cattle, the trait of horn shape exhibits sexual dimorphism (Schafberg & Swalve 2015; Armstong *et al.* 2022). Both ovids (Ibsen 1944) and bovids (Long & Gregory 1978; Aldersey *et al.* 2020) can have scurs, which are horn-like appendages up to 15 cm in length that are not anchored to the skull. However, in modern and intensive animal rearing, horns are perceived to be a welfare issue since they can easily injure other animals (El-Hawari *et al.* 2015; Braun *et al.* 2016) as well as acting as a safety hazard for animal handlers (Rushen *et al.* 2008; ALCASDE 2009). Therefore, international management protocols entail either keeping genetically polled animals, destroying horn-producing cells in young animals (disbudding), or surgically removing horns present in older animals (dehorning; NSW Government 2023). While disbudding and dehorning are common procedures in cattle production systems in Europe, Canada, and the USA (Cozzi *et al.* 2015; Reedman *et al.* 2022), there is generally little need for dehorning or disbudding sheep (Dwyer 2008).

Disbudding is known to fail for various reasons leading to a deformed horn beginning to grow (Sutherland *et al.* 2019; Schoiswohl *et al.* 2022), but unaltered horns may also curve inwards causing the horn tip to penetrate the skin. Forensic examination of ingrown horns has shown long-standing changes to the skin, muscle, and connective tissue, with the formation of scar tissue and a cavity that is deeply infected. These injuries are generally considered to cause the animal unnecessary suffering (Munro & Munro 2008).

The Swedish Food Agency, which provides statistics related to animal welfare notifications flagged by official veterinarians at the country’s abattoirs, received 81 and 90 notifications of ingrown horns in sheep and cattle, respectively, between 2015 and 2022 (The Swedish Food Agency, personal communication 2023); corresponding to an average of ten sheep and eleven cattle with ingrown horns each year. For comparison, a total of 410,000 cattle and 230,000 sheep and lambs were slaughtered in Swedish slaughterhouses in 2022 (Köttföretagen 2023). Any farmer unable to provide a credible and provable explanation for ingrown horns are charged with animal cruelty or violation of the Animal Welfare Act (2018).

### The Swedish judicial system

Violations of the Animal Welfare Act (2018) and Animal Cruelty (Chapter 16 Section 13 in Penal Code) are judged in the district courts, and their decisions may be subsequently taken to the courts of appeal and then to the Supreme Court. The Swedish Animal Welfare Act (2018) became law on July 1st, 2019, and states that an animal that is injured or sick must be given necessary care as soon as possible or be euthanased, and if the injury or illness is so severe that the animal is exposed to severe suffering that cannot be alleviated, the animal must be euthanased (Chapter 4, Section 1). If necessary, care must be provided by a veterinarian or someone else belonging to the group of certified animal health personnel according to the Swedish Law on Activities within Animal Healthcare (2009). This means that, unless they are a qualified veterinarian, the farmer is not allowed to remove the horns. Additionally, surgical procedures, such as disbudding and dehorning, must be performed under anaesthesia. If the farmer fails to provide the animal with the necessary veterinary care, charges may be brought under the Animal Welfare Act (2018), and for animal cruelty if it can be shown that the animal has suffered due to negligence.

Animal cruelty is described as follows in the Swedish Penal Code (Chapter 16, Section 13):

*A person who, intentionally or through gross negligence, improperly exposes an animal to suffering through assault, overexertion, neglect, or in some other way is guilty of animal cruelty, is sentenced to a fine or imprisonment for at most two years. If the offence is severe, the person is guilty of gross animal cruelty and is sentenced to imprisonment for at least six months and at most four years. When assessing whether the offence is severe, particular consideration is given to whether the act:*

1. *Involved serious suffering for the animal or animals;*
2. *Involved a large number of animals; or*
3. *Was otherwise of a particularly ruthless or dangerous nature.*

Gross animal cruelty only became a crime in Sweden after July 1st, 2022. In instances where animal cruelty cannot be proven, the prosecutor may ‘secure’ the prosecution through violation of the Animal Welfare Act (2018) as a secondary or additional charge, i.e. the suffering itself does not need to be proven, only the risk of suffering. Also, if animal cruelty is committed while performing corporate operations, the legal effect may be a corporate fine. Private persons can be sentenced to a conditional sentence, day fines, or imprisonment, if found guilty. Day fines are fixed amounts of money up to 1,000 SEK (€90) to be paid daily as a fine for up to 150 days.

### Aims and research questions

The purpose of this study was to raise awareness about a specific animal welfare problem, i.e. ingrown horns in cattle and sheep, and identify the circumstances under which it occurs. Legislative measures concerning the regular control of horns are missing in Swedish animal welfare legislation, although they are mentioned in the guidelines of official control in abattoirs (SJV 2023b). Ingrown and fractured horns are on the Swedish Food Agency’s list of deviations from animal welfare legislation and the occurrences found at abattoirs are regularly reported to the authorities.

The animal welfare problem is likely to be much larger than is reported to the authorities and ingrown horns are not merely a Swedish problem (Bamaiyi & Turaki 2012; El-Hawari *et al.* 2015; Prasad *et al.* 2016; Government of Western Australia 2018; Queensland Government 2020). Increasing herd sizes (Egger-Danner *et al.* 2020; SJV 2021) are likely to make ingrown horns both more common and more difficult to detect. We therefore looked at legal cases in Sweden where ingrown horns and other horn-related anomalies in cattle or sheep led to prosecutions for violations of the Animal Welfare Act or for animal cruelty, and the following were investigated:

1. The nature of the horn-related anomalies, and which animals were affected;
2. By whom and in what circumstances the horn anomalies were discovered;
3. The reasons given by the accused for not detecting animals with horn-related anomalies or for performing unauthorised dehorning without analgesia; and
4. The results of the legal cases in terms of sentencing and punishment.

## Materials and methods

### Ethical statement

No sensitive personal data according to the GDPR were collected in this study. Court decisions from the legal information services are official documents that are normally anonymised regarding personal identity numbers, even though preliminary investigation reports are not. We have not used any names or places in our report.

### Terminology concerning horns and horn-related anomalies

The term ‘dehorning’ is used differently across countries and jurisdictions. It is sometimes used as an umbrella term to encompass both disbudding and dehorning (Welfare Quality® 2009; SJVFS 2019a,b) and is used synonymously with disbudding in Swedish animal welfare legislation (Animal Welfare Ordinance 2019:66; SJVFS 2022; SJV 2023a), although EU Organic Regulation 2018/848 distinguishes the concepts also in the Swedish translation. Dehorning refers to the removal of the horns after they have formed from the horn bud, occurring at approximately two months of age (Marquette *et al.* 2021). Horn ‘tipping’ or ‘trimming’ is defined as “the removal of the insensitive part of the horn of cattle resulting in a blunt and shorter horn-end” (State of Western Australia 2023).

In the literature, the term ‘overgrown horn’ is also used (El Hawari *et al.* 2015; Prasad *et al.* 2016; Sharma *et al.* 2021). An ‘overgrown horn’ is defined as “maligned shorthorn type horns which curve too far and penetrate the skull, usually into a frontal sinus” (Blood *et al.* 2007). In this article, we use the term ‘ingrown’ to describe horns that have penetrated the skin or other tissue or if the horn touches or puts pressure on the skin or other tissue but has not penetrated it, unless otherwise indicated. The term is also used for cases in which the horn tip (or other part of the horn) is so close to the skin that it requires palpation to examine whether it has reached the skin. We also use the expression ‘horn-related anomaly’ to cover ingrown, fractured, and sawed/cut horns.

### Data acquisition

Notifications regarding horn-related anomalies (ingrown, fractured or otherwise defective horns) from veterinarians in connection with pre-slaughter inspection were received directly from the Swedish Food Agency. The notifications became digitalised in 2015, and no official data are available prior to that year.

The legal cases and associated verdicts were retrieved from legal information services (JP Infonet; https://www.jpinfonet.se/JP-DjurNet) and the preliminary enquiry reports directly from the courts. A search with the terms “horn”, “district court” and “criminal case” (in Swedish) on the JP Djurnet database resulted in 152 hits. The inclusion criterion was that horn-related anomalies (ingrown or fractured horns, or unauthorised dehorning) were the reason for the charge. Administrative court cases where the accused had appealed the County Administrative Board’s decision that the farmer was prohibited from keeping animals after being sentenced for animal cruelty and appeals of the verdicts of criminal cases were excluded. After also excluding duplicates and verdicts not including horn-related anomalies in cattle or sheep, we were left with 32 verdicts from 22 district court (see Table 1).

**Table 1.** Number of notifications about ingrown horns in cattle and sheep sent by abattoirs to the Swedish Food Agency (SFA) from April 2015 to December 2022 and number of verdicts from the Swedish district courts that dealt with cases where horn anomaly led to criminal charges (2008 to 2022)

Year	Number of notifications sent to the SFA (by species)		Number of district courts	Number of verdicts in district courts
	Cattle	Sheep		
2008	–	–	1	1
2009	–	–	–	–
2010	–	–	–	–
2011	–	–	2	2
2012	–	–	2	2
2013	–	–	2	2
2014	–	–	1	3
2015	4	3	1	1
2016	12	15	4	4
2017	17	13	2	2
2018	7	22	4	4
2019	–	–	–	–
2020	17	11	4	4
2021	3	5	3	3
2022	9	12	4	4

NB Notifications concern individual animals while a verdict can involve several animals.

In twelve cases we also used the preliminary enquiry reports for details (e.g. photographs) that were missing in the verdicts, to obtain information relating to our four research questions. The verdicts and the preliminary enquiry reports, where the charges and the evidence are presented, are generally official documents that can be requested from the courts by the public.

The following variables were compiled from district court decisions and preliminary enquiry reports: the year and the name of the district court where the hearing occurred; the time from the discovery of the horn anomaly and the judicial decision; by whom and in what circumstances the horn anomaly was discovered; grounds of prosecution; the number and gender of the accused; whether the accused denied or confessed to the crime; the reasons given for not detecting the horn-related anomalies or for performing unauthorised dehorning; the species, sex and number of animals affected; type of horn-related anomaly; and the verdict and penalty decreed by the court. The Excel® 365 workbook was used for data analysis and to generate descriptive statistics.

## Results

### General aspects

Of the 32 legal cases, seven involved sheep (22%) and 25 involved cattle (78%). In total, the cases involved 38 accused persons and 39 animals, with an average of 2.3 cases per year (range 0–4 cases) between 2008 and 2022. There were no repeat offenders during the evaluated period. Between 2015 and 2022, notifications of ingrown horns at the abattoir to the Swedish Food Agency had been reported between seven and 30 times per year (Table 1). In total, 17 cases (53%) were discovered by animal welfare inspectors during official control activities on the farm, 14 (44%) were discovered by veterinarians during pre-slaughter inspection at abattoirs and one case was discovered by a veterinarian visiting the farm (3%). In 25% of the cases (n = 8), the complaints to county administrative boards came from members of the public. Eight cases (25%) also included other animals (range 1–90 animals of the same or other species) that were neglected. In five of these, the farmers indicated exhaustion, burnout, and illness in the family as the cause of neglect.

Of the accused, three were women (8%) and 35 were men (92%). In two sheep cases, women were prosecuted, while in one cattle case, a woman was prosecuted jointly with a man. One of the accused men was an animal transporter, and the rest were farmers or farm employees. In 12 (38%) of the legal cases the accused were indicted for animal cruelty, with violation of the Animal Welfare Act (2018) as a secondary charge, solely for animal cruelty in 18 cases (56%), and solely for violation of the Animal Welfare Act (2018) in two cases (6%). In the six legal cases with two accused (19%), both were charged with having jointly committed the crime. In one case, both the farmer and the transporter were charged with letting the injured animals be transported. The time from the discovery of the horn anomaly and the judicial decision was a maximum of two years in 26 cases (81%), but in five cases (16%), it took three years, and in one (3%), it took > 4 years. Reasons for acquittals included insufficient charge descriptions, insufficient evidence of how the horn-related anomaly occurred, and insufficient evidence that the animal(s) were exposed to pain and suffering. None of the 26 convictions resulted in imprisonment, but the results did include conditional sentencing (22%) and/or a day fine (84%). The number of day fines varied from 30 to 100 (the size of the day fine is dependent of the defendant’s income). Corporate fines varied from 10,000 SEK (~€900) to 150,000 SEK (~€13,500). In one case, the district court found that an unusually long time (3–4 years) had passed between the crimes being committed and the accused being sentenced, which the district court considered in sentencing. The penalty was therefore determined to be a conditional sentence combined with 30-day fines.

**Figure 1.**
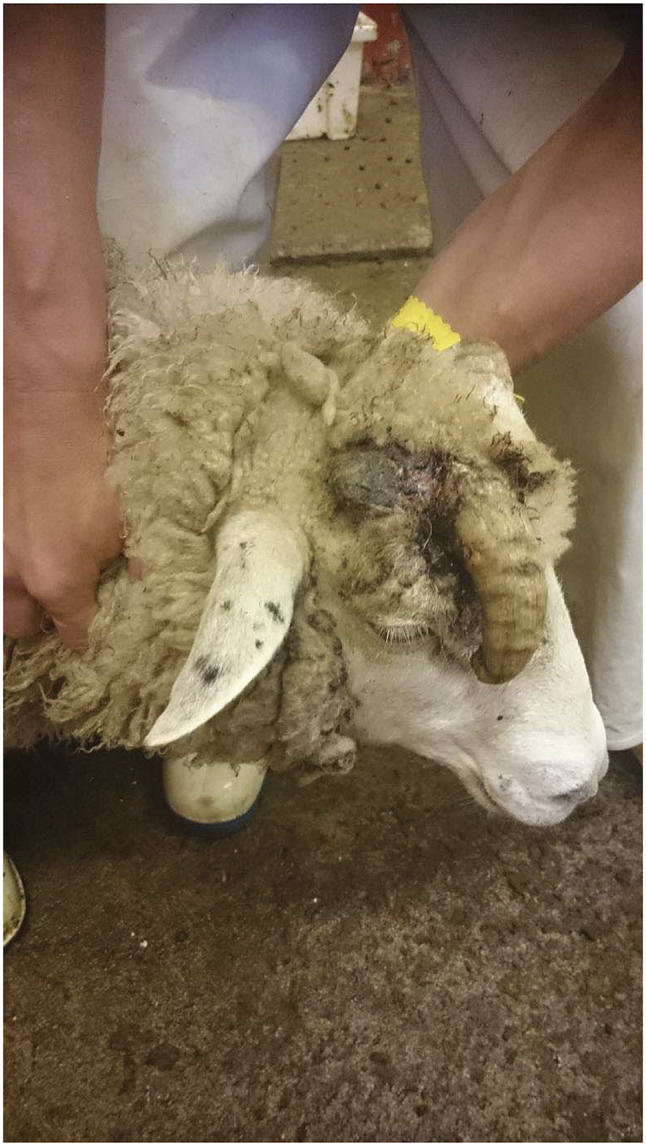
Preslaughter inspection at an abattoir. The right horn of a ram is fractured at the base and hangs on his face. (Photograph courtesy of The Swedish Food Agency).

**Figure 2.**
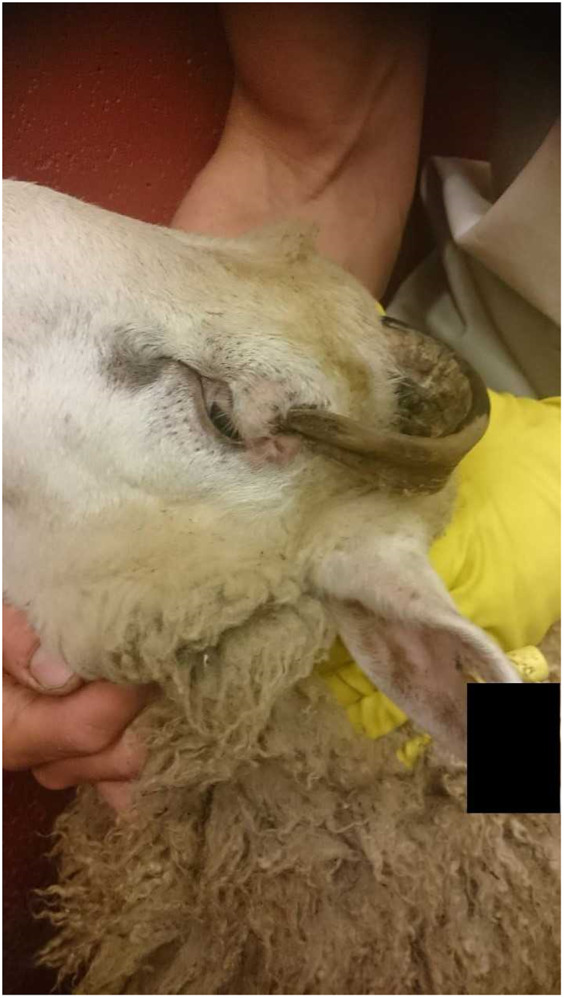
Preslaughter inspection at an abattoir. The left horn of a ram exerts pressure on his eyelid, which is crusted and swollen. The verdict of the district court resulted in acquittal from the charges of animal cruelty. (Photograph courtesy of The Swedish Food Agency).

In the seven legal cases involving sheep, all nine rams had incurred injuries, ranging from the horn touching or penetrating soft tissues (eye or skin) and fractured horns. In one case, the veterinarian made the decision to immediately euthanase the ram on finding that the horn had penetrated the ram’s eyeball by 1 cm. In another case the court stated since the two rams in question did not have long left to live, it would not be necessary to trim the horns prior to sending them to the abattoir, despite both suffering injuries to innervated tissues (chronic inflammation in an eyelid due to pressure from the horn in one animal and a fractured horn in the other; Figures 1 and 2). The accused was cleared of all charges. Finally, in another case, both horns of the ram were ingrown, and an autopsy from the National Veterinary Institute showed that the right horn had penetrated several types of tissue causing extensive damage (destruction) due to pressure on the frontal bone near the eye. The observed damage to the bone tissue was estimated to have lasted for several weeks since the reshaping and breakdown bones takes time. The left horn also grew towards the head and touched the skin but was yet to penetrated it. An expert veterinary witness had seen several cases in which a horn was very close (but not touching) the skin creating favourable conditions for fly larvae to infest the wound created between the horn and the head. The prosecutor did not include the left horn in the charge or refute the farmer’s claim that this sheep breed tends to be hornless with only a small risk of ingrown horns occurring. The accused was cleared of both charges.

### Dissenting opinions of the lay judges

In four (16%) legal cases concerning cattle, the lay judges had dissenting opinions when making the judicial decision. For example, a lay judge stated that because the ingrown horn was difficult to detect and the farmer had many animals to look after, he had not failed in his supervision of the animal. In another case, a lay judge stated that because the steer did not display any external signs of being unwell, e.g. emaciation, the farmer had no reason to pay specific attention to the steer. In addition, because this farmer had many animals to look after, the lay judge did not find that the farmer had failed in his supervision of the steer and, therefore, the case should be dismissed.

In one case, two lay judges considered that the cow in question had been subject to suffering for an extended period and such were the circumstances that a fine would be insufficient punishment. They argued that the penalty should therefore be a conditional sentence in combination with a fine. In another case, the tip of the horn was uneven and deformed, which gave an indication that the horn had been in contact with the skin for a long time and was therefore unable to grow naturally. A lay judge considered the defendant to have caused animal suffering through gross negligence and should therefore be sentenced for animal cruelty.

### The nature of the horn-related anomalies, and types of animals affected

Horn-related anomalies were more common in males for both sheep (100%) and cattle (63%). Four rams (44%) had at least one horn that had penetrated either the skin or one eye, six (67%) had at least one horn pressing against the skin or cornea of one eye, and one had a fractured horn (11%). An animal could have more than one horn-related anomaly. Extreme injuries were found in two of the nine sheep (22%): one ram had a horn that had penetrated the eyeball (~1 cm) while another showed a depression in the processus zygomaticus of the frontal bone as a result of pressure from the growing horn.

**Figure 3.**
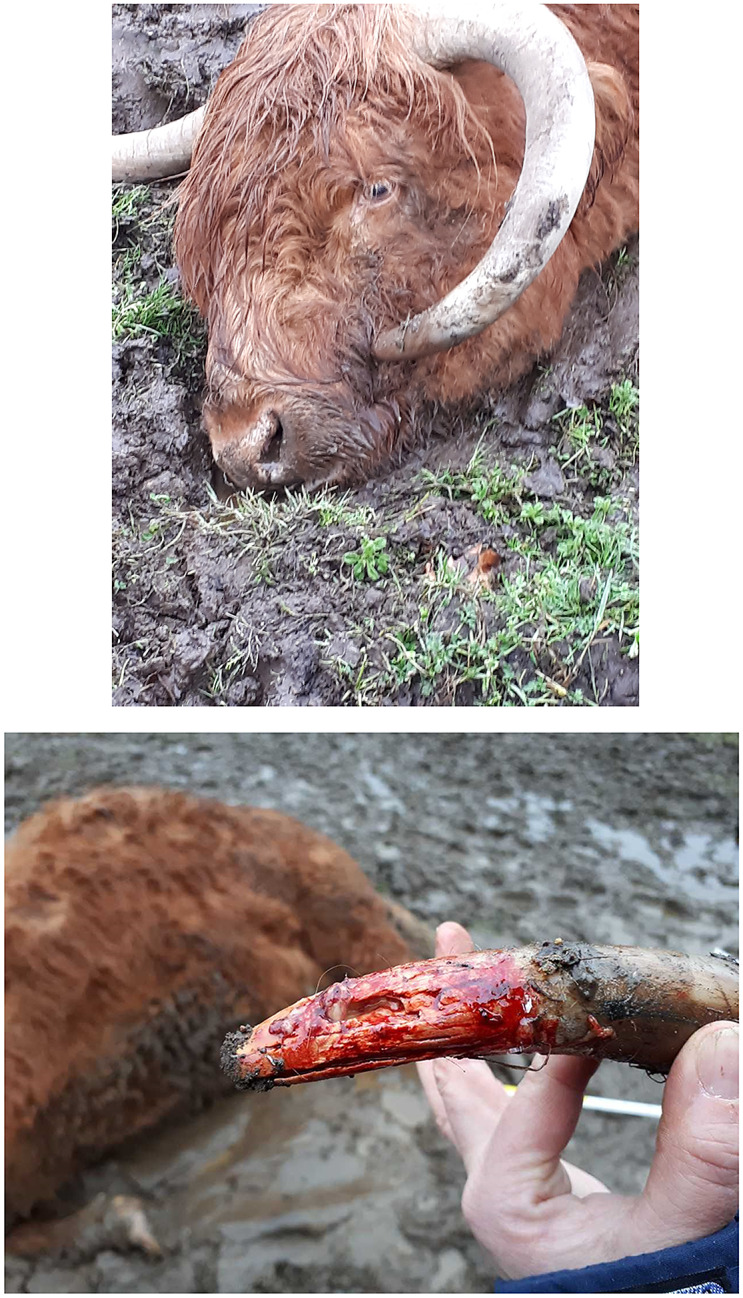
Showing (a) a Highland cow with an ingrown horn and (b) the bloody part of the horn that had grown through the cow’s cheek into her mouth cavity. It appeared that she had chewed the horn since its surface showed damage, and there was an accumulation of pus in the sore. The prosecutor could not show that the horn was ingrown, as a witness thought that the injuries could have been caused by trauma. Since the crime description did not include the possibility of trauma, the charge was dismissed. The verdict of the district court resulted in acquittal from the charges of animal cruelty. (Photographs courtesy of The Swedish Food Agency).

In 25 cases, 30 cattle (19 males, of which eleven were castrated; ten females, one unknown sex) were affected by horn-related anomalies. Two were Highland cows and one was a dairy cow. In 19 of 30 cattle (63%), a horn had penetrated the skin and underlying tissue; in eight of these the penetration depth was 1–3 cm, and in two, it was > 3 cm. In one of these, the tip of the right horn had penetrated 6-cm into the sinus frontalis. This process was estimated to have taken 6–12 months to occur, and the suffering assessed as severe. Three cattle had horns pressing onto the skin, and two cattle horns pressed on the cornea or an eyelid. Two cattle were dehorned without analgesia and two others had fractured horns. In one steer, both horns had penetrated through the periosteum into the skull bone, and in one Highland cow, the horn had penetrated 7 cm through the cheek into the mouth cavity (Figure 3[a]).

### By whom and in what circumstances horn anomalies were discovered

Fifty-three percent of the legal cases (n = 17) were brought following discovery by animal welfare inspectors during official animal welfare control activities, either at routine scheduled visits or at official visits following a complaint. It was not always clear who had lodged the complaint to the county administrative boards, but in at least eight cases (25%), they arose from the public (e.g. dog walkers and bird watchers) and, in one case, from a farm employee. Fourteen cases (44%) were discovered by official veterinarians at the abattoir, while one (3%) was reported by a veterinarian who discovered an ingrown horn during a farm visit.

### Reasons given by the accused

Animal-based reasons for failing to detect horn-related anomalies at the farm included that the animals grew and behaved normally, that the animals were long-haired, shy or hard to reach, that the horn had not grown into the soft issue but had penetrated it after a sudden horn fracture as a result of fighting or transportation, or that the horn-related anomaly had occurred elsewhere. Human-based reasons included heavy workload, illness or personal/family problems, poor knowledge regarding ingrown horns or a lack of awareness that it is illegal to dehorn animals without analgesia.

In all but one legal case concerning sheep (86%), the owner denied the crime and the presence of horn-related anomalies in the sheep on their farm. In three cases (43%), the owners indicated that the rams must have been fighting immediately prior to or during transportation to the abattoir and that the horn had been knocked or pushed in. In one case (14%), the owner admitted that he was unaware horns could grow in at all, and in two others (29%) the owners claimed that the sheep could not have developed ingrown horns and experienced suffering since they were fat and had grown well.

In twelve of the legal cases concerning cattle (48%), the accused indicated that the ingrown horns occurred due to failed attempts at disbudding and in seven cases (28%), the farmer had observed the horn anomaly but misjudged how fast horn growth could occur. In another case, the farmer stated that four of his 200 animals had deformed horns due to “incomplete burning” during disbudding and that he had been able to insert a finger between the horn and skin as recently as ten or 12 days before the bull went to slaughter, and he could not explain how the horn had ended up as it did in the photographs. One farmer expressed that according to the abattoir personnel, it is common for animals to have ingrown horns, but “it is taboo, and nobody talks about it”.

In four cases, the accused and their witnesses argued it to be possible for a horn to become knocked deeper into an animal’s head when, for example, it falls, fights or hits itself. In another case, however, two veterinary witnesses found such an explanation less credible. They argued that when a horn fractures due to outer force, it causes profuse bleeding and that the horn becomes loose.

### Results of the legal cases in terms of sentencing and punishment

Of the 38 accused, 34 pleaded not guilty (90%), two confessed to animal cruelty (5%) and two confessed to violating the Animal Welfare Act (5%). Seven of the accused (18%) were acquitted, and 31 (82%) convicted of the crime. Two of the three accused women (67%) and four of the 35 accused men (11%) were acquitted by the district courts. In cases with two accused persons, both were either jointly convicted or acquitted. Reasons for acquittals included insufficient charge descriptions, insufficient evidence of how the horn-related anomaly occurred and insufficient evidence that the animal(s) were exposed to pain and suffering. None of the 26 convictions resulted in imprisonment, but conditional sentences (22%) and/or a day fine (84%) were imposed. In three of the legal cases (9%), all concerning cattle farms, the accused were sentenced to pay corporate fines in addition to the day fine. In one case regarding another cattle farm, there was a corporate fine only. In one legal case concerning two injured rams, the district court ruled that providing care had been unnecessary since they were shortly due to be slaughtered. In four cattle cases, the lay judges expressed dissenting opinions concerning the judicial decision: two wishing to acquit the accused while two sought a conviction.

## Discussion

### General aspects

Our study addresses a very specific animal welfare problem that is important because it involves the pain and often the prolonged suffering of the animals in question. The results show that passive surveillance and self-control programmes are insufficient in detecting these types of welfare issues and that it is important to maintain official animal welfare controls at abattoirs and on farms.

In Sweden, a total of 1,415 animal cruelty convictions were issued by the District Courts between 2008 and 2021 (Brå 2023), resulting in an average of 101 convictions per year; convictions for ingrown horns constitute less than three percent of these. There are no data available on the proportion of production animals among these cases.

### Reasons given by the accused for not detecting ingrown horns

Health issues, exhaustion and burnout were given as reasons for not detecting ingrown horns in the five legal cases where entire herds were neglected. In Finland, health, economic problems and excessive numbers of animals are common justifications for animal welfare crimes (Väärikkälä *et al.* 2020). Several of the accused (and some lay judges) claimed that their animals could not have been suffering because they were fat and well-grown, and that suffering animals do not eat. According to Munro and Munro (2008), this statement is a common defence against alleged neglect of lame farm animals, but it is not supported by observations of injured animals; even animals with long-standing and painful conditions can remain in fair or good bodily condition. Furthermore, the phenomenon of ingrown horns is a recognised medical condition, and ruminants do show signs of pain. Rams and goats have shown increased circling and head tilting in cases of unilateral overgrown horns and dullness and apathy in bilateral overgrown horns (El-Hawari *et al.* 2015). Changes in behavioural expression and body posture have been shown in 6–8 week old lambs that were subjected to painful husbandry procedures (Grant *et al.* 2020). Signs of pain in cattle can be seen in their ear, head and back positions, as well as their in facial expressions, and general alertness levels (Gleerup *et al.* 2015). For example, upon arrival at the abattoir, a cow with a horn ingrown into its skull bone showed signs of apathy and was immediately euthanased by the veterinarian. In another case, a bull was seen at one point touching the building with its damaged horn and then immediately recoiling. When the veterinarian examined one of the cows with ingrown horns, she saw that the cows showed touch aversion of their heads, indicating they were in pain. Ingrown horns cause severe, protracted, and unnecessary suffering for the affected animals, as stated by the Swedish Board of Agriculture (SJV 2011).

### Expert and witness statements

In specialised areas of criminal law, expert witnesses are needed to explain special circumstances and to refute implausible claims expressed by the accused. In one case, the district court found evidence from a veterinary expert witness to be insufficient because he had not seen the affected animal in person and based his findings on documents and photographs. The court chose therefore to believe the testimony of the keeper and his two witnesses who had stated that the horn was not ingrown but been knocked into the animal’s head due to external force.

In a case involving a Highland cow, the prosecutor could not show that the horn was ingrown, as an expert witness thought that the injuries seen could also have been caused by trauma. The charge was dismissed even though the case photographs (Figures 3[a],[b] offered clear evidence that the horn was not fractured and was still attached to the skull.

One farmer claimed that the breed in question lacked horns in the true sense and only had small buttons that fall off. The prosecutor could have checked this claim and referred to the breed standard which states that “a developed horn base or small horns are the ideal. Heavy horns are undesirable but permissible” (Dorper 2020).

### Distribution of legal cases between the district courts

A disproportionate number of legal cases (eight of 32 cases) were handled by one of the 48 district courts in Sweden. This may be explained by several factors. First, the competent court for criminal cases is the court of the place where the offence was committed (Chapter 19, Section 1, Swedish Code of Judicial Procedure) and this district court is located in a county that contains agricultural holdings with large animal stocks (Jordbruksverket 2020). As the horn-related anomalies were first detected during pre-slaughter inspections by an official veterinarian in four of eight legal cases, the high number of legal cases in this district court could also be explained by the county containing one of Sweden’s largest abattoirs. In 2021, approximately 12.5% of the nation’s culled cattle and 9.6% of the sheep were handled by this operation (SJV 2023a). However, a district court located in another county containing an abattoir that slaughtered approximately twice as many cattle and four times as many sheep did not handle any legal cases concerning ingrown horns from abattoirs. This suggests either that the diligence in reporting these types of injuries differs between regions or that different prosecutors are more or less likely to prioritise these legal cases. A third reason could be that the number of cases may depend on the effectiveness of official control of animal welfare by the County Administrative Boards and the effectiveness and priorities of the judiciary in investigating and, if necessary, prosecuting the crimes. It is possible that the crimes are time-barred as they have been deprioritised by the justice system.

### Sheep

In total, there were approximately 8,500 farms with sheep in Sweden in 2021, and the average herd size was 32.1 adult animals (SJV 2021). The larger the herd, the more time-consuming the daily supervision of the animals and if animals are grazing on large pastures, there is a risk of neglecting certain animals. However, only 14% of the Swedish farms had herds larger than 49 adult animals. In the legal cases concerning sheep, the herd sizes were > 49 animals in four cases, 25–35 animals in one case, 15 animals in one case and an unknown size in another case.

### Cattle

In several cases, mention was made of animals being disbudded as calves on the farm and the procedure occasionally failing. For example, a farmer argued that disbudding and castration of the calves were performed at the same time and that the disbudding had been unsuccessful due to a defective cautery instrument. The Swedish Board of Agriculture estimated that 5% of disbudding operations fail (SJV 2011). If 70,000 animals are disbudded yearly in Sweden by the farmer association Växa Sverige (Svenberg 2020) at least 3,500 animals are at risk of developing ingrown horns each year.

**Figure 4.**
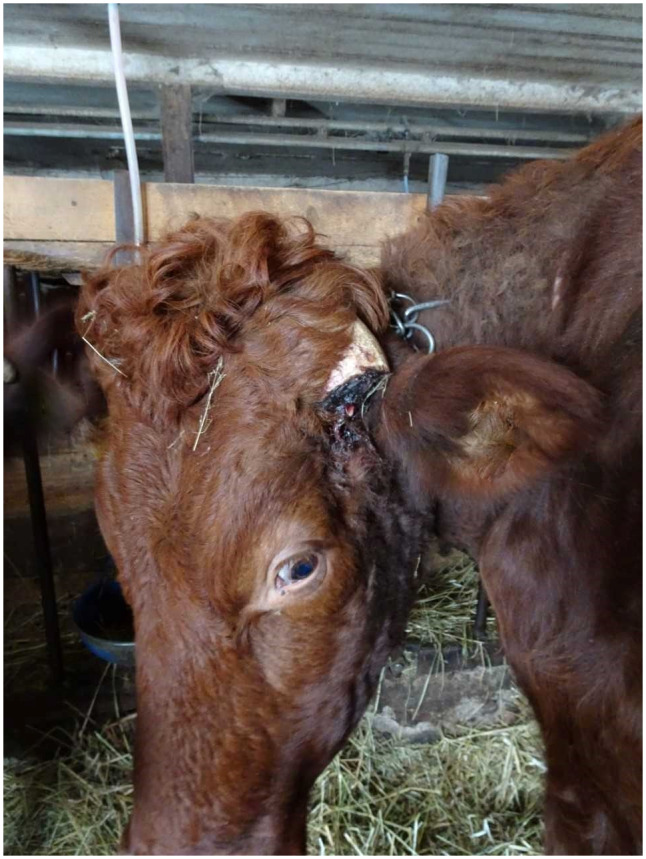
Horns have been sawn off so deeply that the horn sinus was exposed and the action performed without analgesia or proper bandaging. The verdict of the district court resulted in conviction for animal cruelty. (Photograph courtesy of The Swedish Food Agency).

In two cases, the accused had sawn off the ingrown horns at such a depth that the horn sinus was opened and done so without using analgesia or proper bandaging (Figure 4). Dehorning so close to the horn base was noted to cause profuse bleeding and be extremely painful, taking as long as three months to heal.

### Final judgements and penalties

Six of the verdicts (19%) were acquittals (five men and two women) and 26 (81%) were convictions (30 men and one woman). Ninety-six percent of the animal welfare violations in Finland concerning cattle and pig welfare resulted in convictions (n = 189) and inconsistent evidence was the reason for withdrawal in one case (1%; Väärikkälä *et al.* 2020).

None of the crimes were deemed serious enough by courts to warrant a custodial sentence even although in certain instances it was entire herds that were subjected to suffering not simply individual(s) with horn-related anomalies. It is very rare for imprisonment to be imposed as a punishment for animal cruelty in Sweden, and when it does happen, the victim is usually a companion animal, most often a dog. To our knowledge, the longest ever Swedish prison sentence for animal cruelty is one year and three months, for a torture-like abuse of a dog (SOU 2020). This difference in legal propensity in judging different species of animals does not reflect legal differences but more judges’ and jurors’ view of companion animals being more protectable than production animals. According to Sinclair *et al.* (2022) in some countries, the welfare of dogs was considered more important than human welfare.

Comparing punishments is challenging given the differences in the crime descriptions. Among the current cases, conditional sentences (22%) and/or a day fine (75%) were the most stringent punishments handed out.

As several findings were made at abattoirs, the animals had been subjected to transportation. The transporter has a strict responsibility to deny the loading of animals with defects or injuries. Even if there are, to our knowledge, no guidelines that address the issue of ingrown horns and fitness for transport in the relatively vague EU legislation, it should be clear to every animal transport professional that loading an animal with ingrown horns for slaughter is not permitted because the animal is unfit for transport. In New Zealand, for example, this is clearly stated in the Animal Welfare (Care and Procedures) Regulations 2018 (Part 1, Clause 38, Subclause 1-2). Despite these legal demands, none of the transport drivers in the studied reports had noticed injured animals or animals with ingrown horns during loading or unloading.

### Study limitations

It is possible that we missed legal cases in our search and that the cases addressed here are only a proportion of the existing cases. It is also important to note that we did not check the appeals and that the rulings in the district courts may have been changed in the court of appeals. Furthermore, the actual number of violations is probably much higher due to cases remaining unreported, being closed, or never being investigated by the authorities. Since the personal identity numbers were masked in the legal cases, it was not possible to define the age categories of the accused persons. The amount of information in the verdicts varied and, for example, it was not always clear whether the inspection was unannounced or whether it was a routine visit. It was also not always clear who reported ingrown horns to the authorities, only that the official control of animal welfare took place because a report was received.

Only one legal level of animal cruelty in the Swedish penal code was engaged for the cases we have discussed. Since ingrown horns develop over an extended period of time, we would argue that this type of injury is more in keeping with the new crime category of gross animal cruelty that was added to the Swedish criminal code on July 1st, 2022. A conviction would then lead to a minimum of six months of imprisonment.

### Animal welfare implications

Prophylactic measures to find ingrown horns must be included in everyday handling routines. Caretakers of horned animals should inspect horns on individual animals routinely and frequently to enable interventions long before injuries to nerve-rich tissue occur.

### Concluding recommendations

The inspection of horns should be included as a crucial component of the regulations on the keeping of horn-bearing animals, in self-control programmes, and in control authorities’ checklists. A horn growing in the direction of the animal’s head must be addressed long before it reaches tissue to avoid discomfort, larval infection, pain, and suffering. Since ruminants make constant use of their facial muscles during rumination, the distance between a horn and the skin at rest and at rumination can vary considerably. Animals with black heads and long hair require particularly careful examination.

Terminology regarding horns in the Swedish animal welfare legislation should undergo clarification with differences between various interventions, e.g. disbudding, tipping/trimming and dehorning made explicit, as should which individuals are authorised to perform such procedures.

Early slaughter of sheep and cattle with severe horn-related anomalies could be a better option for growing animals that otherwise must be dehorned, as the dehorning procedure can be painful and stressful for the animal and reduces the subsequent risk of horn growth being misjudged.

Prosecutors in Sweden should always use crime against the Animal Welfare Act as a secondary charge and research is needed to investigate how often disbudding fails, and how horn growth develops after a partially successful disbudding.
